# Effects of exercise training with short‐duration intermittent hypoxia on endurance performance and muscle metabolism in well‐trained mice

**DOI:** 10.14814/phy2.14182

**Published:** 2019-07-21

**Authors:** Junichi Suzuki

**Affiliations:** ^1^ Laboratory of Exercise Physiology, Health and Sports Sciences, Course of Sports Education, Department of Education Hokkaido University of Education Iwamizawa Hokkaido Japan

**Keywords:** Dynamin‐related protein‐1, exercise training, fatty acid metabolism, intermittent hypoxia, PGC‐1*α*

## Abstract

The author previously reported that short‐duration intermittent hypoxia had additive effects on improvements in endurance capacity by enhancing fatty acid metabolism. The present study was designed to investigate the effects of short‐duration intermittent hypoxia on endurance capacity, metabolic enzyme activity, and protein levels associated with mitochondrial biogenesis in well‐trained mice. Mice in the training group were housed in a cage with a running wheel for 7 weeks from 5 weeks old. Voluntary running markedly increased maximal work values by 5.0‐fold. Trained mice were then subjected to either endurance treadmill training (ET) for 60 min or hybrid training (HT, ET for 30 min followed by sprint interval exercise (5‐sec run‐10‐sec rest) for 30 min) with (H‐ET or H‐HT) or without (ET or HT) short‐duration intermittent hypoxia (4 cycles of 12–13% O_2_ for 15 min and 20.9% O_2_ for 10 min) for 4 weeks. Maximal endurance capacity was markedly greater in the H‐ET and H‐HT than ET and HT groups, respectively. H‐ET and H‐HT increased activity levels of 3‐hydroxyacyl‐CoA‐dehydrogenase in oxidative muscle portion and pyruvate dehydrogenase complex in glycolytic muscle portion. These activity levels were significantly correlated with maximal endurance capacity. Protein levels of dynamin‐related protein‐1 were increased more by H‐ET and H‐HT than by ET and HT, but were not significantly correlated with maximal work. These results suggest that intermittent hypoxic exposure has beneficial effects on endurance and hybrid training to improve the endurance capacity via improving fatty acid and pyruvate oxidation in highly trained mice.

## Introduction

Hypoxic exposure has been used in the training regimens of elite endurance athletes in order to enhance endurance capacity at sea level. A large number of studies have investigated the benefits of hypoxic exposure on sea‐level performance (Terrados et al. 1990; Levine et al. 1992; Vogt et al. 2001; Julian et al. 2004; Katayama et al. 2004; Bakkman et al. 2007; Vogt and Hoppeler 2010; De Paula and Niebauer 2012). However, an essential strategy to improve athletic performance has not yet been established.

Chronic hypoxic exposure was shown to increase glycolytic enzyme activity and, in contrast, to decrease lipid oxidation in order to survive in a low oxygen atmosphere (Kennedy et al. 2001). Under hypoxic conditions, hypoxia inducible factor (HIF)‐1*α* is stabilized and translocated to the nucleus, thereby up‐regulating HIF‐responsive genes (Maxwell et al. 1999). Chronic stabilization of HIF‐1*α* inhibited fatty acid oxidation by reducing peroxisome proliferator‐activated receptor *α* (PPAR*α*) (Belanger et al. 2007) and peroxisome proliferator‐activated receptor gamma coactivator 1‐alpha (PGC‐1*α*) expression in vitro (Slot et al. 2014). This may be one of the reasons why effects of chronic hypoxic training, that is, exercise under hypoxia, on endurance performance are controversial. Normoxic training with several hours of intermittent hypoxic exposure at rest may attenuate these negative effects of HIF‐1*α* on endurance performance.

Repeated short‐duration hypoxic/normoxic exposure has been developed to alleviate the physical and mental stresses induced by long‐term hypoxic exposure. However, the effects of a 3–6‐min hypoxia/normoxia cycle on exercise performance in humans remain controversial (Julian et al. 2004; Hinckson et al. 2007; Bärtsch et al. 2008; Bonetti et al. 2009; Mekjavic et al. 2012). Repeated hypoxic exposure, comprising a slightly longer hypoxia/normoxia cycle, was shown to improve exercise performance in rodents. Normoxic exercise training in combination with short‐duration intermittent hypoxia at rest (5 cycles per day of 12% O_2_ for 15 min and 20.9% O_2_ for 15 min) for 2 weeks markedly improved the swimming time to exhaustion and maximal oxygen consumption in rats (Drevytska et al. 2012). Running exercise under normoxia with short‐duration intermittent hypoxia (4 cycles per day of 12% O_2_ for 15 min and 20.9% O_2_ for 10 min) for 3 weeks markedly improved exercise performance by enhancing 3‐hydroxyacyl‐CoA‐dehydrogenase (HAD) activity and mRNA expression of PGC‐1*α* in mice (Suzuki 2016). In that study, exercise training was conducted for only 3 weeks using untrained adult (12‐week‐old) mice. Athletes are more likely to begin their athletic career in their childhood and continue to carry out a daily training regimen throughout their career. In order to clarify whether intermittent hypoxic exposure has beneficial effects to these highly trained individuals, experiments need to be performed using highly trained animals. Thus, the present study was conducted to examine whether normoxic exercise training with intermittent hypoxia alters enzyme activity levels and protein expression levels associated with mitochondrial biogenesis in mice that have been trained from an early age.

PGC‐1*α* has central roles in the regulation of skeletal muscle adaptation in response to exercise training (Wu et al. 1999; Vega et al. 2000). High‐intensity interval exercise (6 × 30‐sec all‐out cycling) followed by endurance exercise (60% VO_2_max for 60 min) markedly enhanced mRNA levels of PGC‐1*α* compared with those observed after respective, single, uncombined regimens in trained human muscle (Skovgaard et al. 2016). Thus, hybrid exercise training (HT) consisting of interval and endurance exercise with intermittent hypoxia may be beneficial to improve the endurance capacity of highly trained individuals.

Endurance exercise has been shown to alter mitochondrial fusion and fission in skeletal muscle. Acute endurance exercise attenuated mitofusion (Mfn)‐1 protein levels (Ding et al. 2010), while it increased phosphorylation levels of the mitochondrial fission marker dynamin‐related protein (Drp)‐1 in rodent skeletal muscle (Pagano et al. 2014). However, the expression of these marker proteins after chronic exercise training with intermittent hypoxic exposure has yet to be clearly demonstrated.

In the present study, experiments were designed to elucidate the effects of endurance exercise or hybrid exercise with short‐duration intermittent hypoxia on the endurance capacity as well as metabolic enzyme activity levels and the expression of proteins involved in mitochondrial biogenesis in well‐trained mice.

## Materials and Methods

### Ethical approval

All procedures were approved by the Animal Care and Use Committee of Hokkaido University of Education and performed in accordance with the "Guiding Principles for the Care and Use of Animals in the Field of Physiological Sciences" of the Physiological Society of Japan and the “European Convention for the Protection of Vertebrate Animals used for Experimental and other Scientific Purposes” (Council of Europe No. 123, Strasbourg, 1985).

### Animals

Fifty male multi‐cross hybrid mice (4 weeks old) were purchased from Clea Japan Inc. (Tokyo, Japan) and housed under the conditions of a controlled temperature (24 ± 1°C) and relative humidity of approximately 50%. Lighting (7:00–19:00) was controlled automatically. All mice were given commercial laboratory chow (solid CE‐2, Clea Japan) and tap water ad libitum. After mice had been fed for 1 week and allowed to adapt to the new environment, they were randomly assigned to a sedentary control group (Sed, *n* = 10) or training group (*n* = 40). Mice in the training group were individually housed in a cage with a wheel activity device (13 cm in diameter) for 7 weeks. Wheel activity (distance and running time) was monitored and recorded using digital bike computers (CC‐VL820, Cateye Co., Ltd., Osaka Japan). Mice in the Sed group were housed individually throughout the experiment. To familiarize mice with a treadmill device, all mice including those in the Sed group were subjected to walking once a week on a controlled treadmill (Modular motor assay, Columbus Instruments Inc., Columbus, OH, USA) for 3 min per day at 10–15 m min^−1^ with a 5‐deg incline.

**Figure 1:**
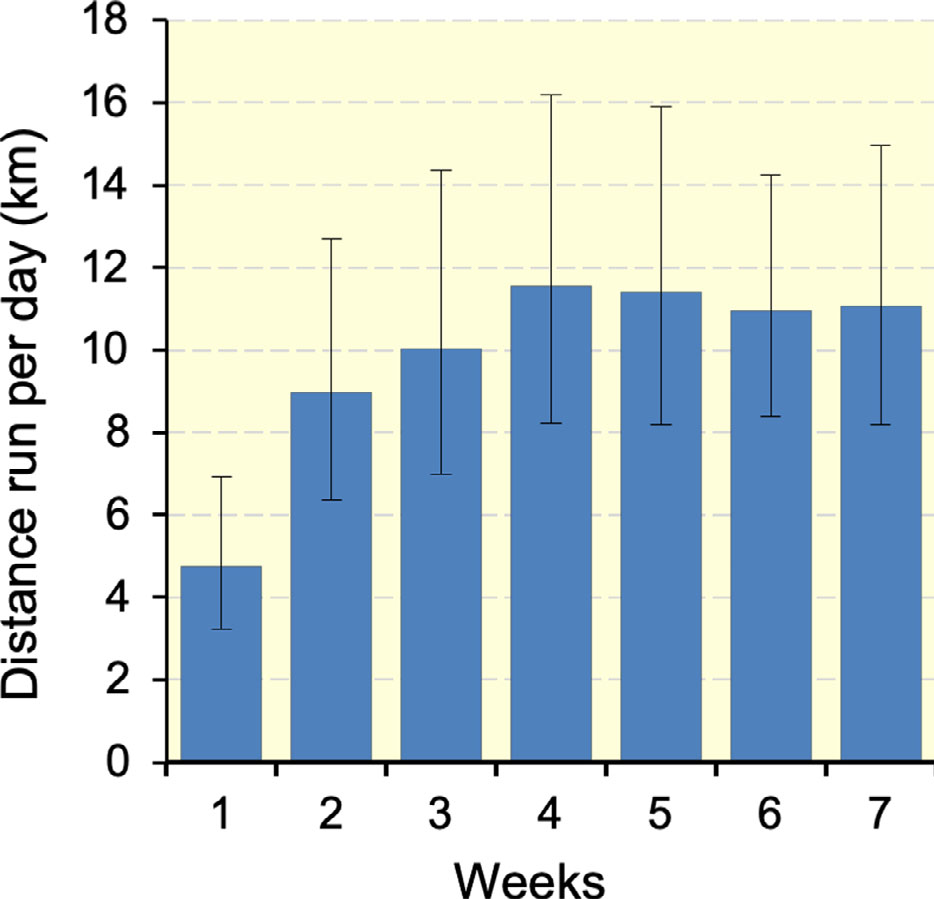
Distance run per day during voluntary wheel training. Values are presented as means ± SD.

The running distance during voluntary wheel training is shown in Figure 1. Following the training, mice were given a 48‐h non‐exercise period prior to the maximal exercise capacity test. The test was performed with a graded ramp running protocol using the controlled treadmill as reported previously (Suzuki 2019). Total work (joule) was calculated by the product of body weight (kg), speed (m sec^−1^), time (sec), slope (%), and 9.8 (m sec^−2^). Exhaustion was defined when the mouse stayed for >5 sec on the metal grid (no electrical shock) at the rear of the treadmill, despite external gentle touching being applied to the tail with a bamboo stick (0.8 mm in diameter).

**Figure 2:**
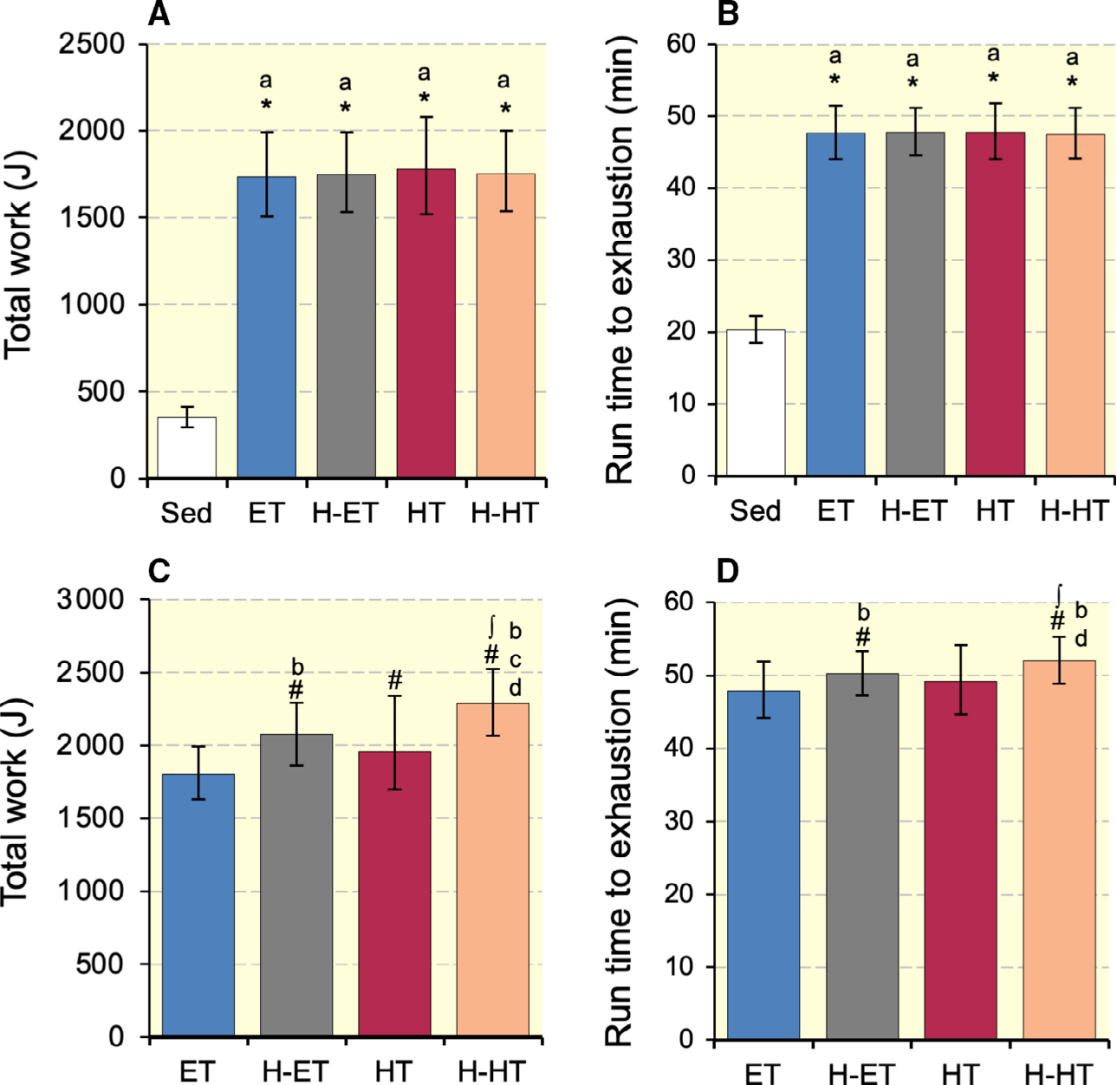
(A and C) Total work in endurance capacity test after 7 weeks of voluntary wheel running and after 4 weeks of treadmill exercise training, respectively. (B and D) Run time to exhaustion values after 7 weeks of voluntary wheel running and after 4 weeks of treadmill exercise training, respectively. Sed, sedentary control group; ET, endurance‐trained group; H‐ET, intermittent hypoxia with endurance‐trained group; HT, hybrid‐trained group; H‐HT, intermittent hypoxia with hybrid interval‐trained group. #, Significantly different from pre‐treadmill training values of each group shown in panel A or B at *P* < 0.05 using Student's paired *t*‐test. Significant differences from the * Sed and ∫ ET groups at *P* < 0.05 using the Tukey‐Kramer multiple comparisons test. Values are presented as means ± SD. a, b, c, and d, the 95% confidence interval did not contain the parameter value specified in the null hypothesis between the Sed, ET, H‐ET, and HT groups, respectively.

After the test, mice in the training group were divided into an endurance‐trained group (ET, *n* = 10), intermittent hypoxic exposure with endurance‐trained group (H‐ET, *n* = 10), hybrid‐trained group (HT, *n* = 10), or intermittent hypoxic exposure with hybrid‐trained group (H‐HT, *n* = 10) so as to match the mean and SD values of total work (Fig. 2A).

The intermittent hypoxic exposure groups were subjected to intermittent hypoxic exposure, which consisted of four cycles of hypoxic air (13% O_2_ for the first 2 weeks and 12% O_2_ for the latter 2 weeks) breathing for 15 min and room air (20.9% O_2_) breathing for 10 min. Exposure was once every day, 6 days per week for 4 weeks. All experimental procedures were done in the laboratory at an altitude of 41 m above sea level.

All treadmill exercise procedures were conducted under normoxic conditions. Mice in the training groups were subjected to exercise training for 4 weeks, 6 days per week using a rodent treadmill (KN‐73, Natsume Co., Tokyo, Japan). For ET, mice ran for 60 min at 20 m min^−1^ with a 15‐deg incline on the first and second days of training. The running speed was increased to 25 and 27.5 m min^−1^ on the first and fourth days of the second week, respectively, and to 30 m min^−1^ on the fourth day of the third week. For HT, mice ran for 30 min of endurance exercise as described above and were subjected to an interval exercise regimen (5‐sec run‐10‐sec rest) for 30 min interposed by a 5‐min rest. In the interval exercise regimen, mice ran at 30 m min^−1^ with a 15‐deg incline on the first day of training. The running speed was increased to 35, 37.5, 40, and 42.5 m min^−1^ on the 4th, 8th, 11th, and 15th days of training, respectively. The maximal exercise capacity test was performed 48 h after the last run, as described above.

Forty‐eight hours after the exercise capacity test, mice were anesthetized with *α*‐chloralose (0.06 g kg^−1^) and urethane (0.7 g kg^‐1^) by intraperitoneal injection. A toe pinch response was used to verify appropriate anesthesia. The left soleus (SOL), plantaris (PL), and gastrocnemius muscles were excised, and both lateral and medial deep red regions (Gr) of the gastrocnemius were isolated from the superficial white region (Gw). The diaphragm (DIA) was excised. All samples were frozen in liquid nitrogen for biochemical analyses. The corresponding muscles on the right side were excised and placed in embedding medium, OCT compound (Miles Inc., Elkhart, IN), and then rapidly frozen in isopentane cooled to its melting point (−160°C) with liquid nitrogen. Mice were killed by excision of the heart. The heart was weighed, and frozen in liquid nitrogen. All tissue samples were stored at −80°C until analyses.

### Histological analyses

**Figure 3:**
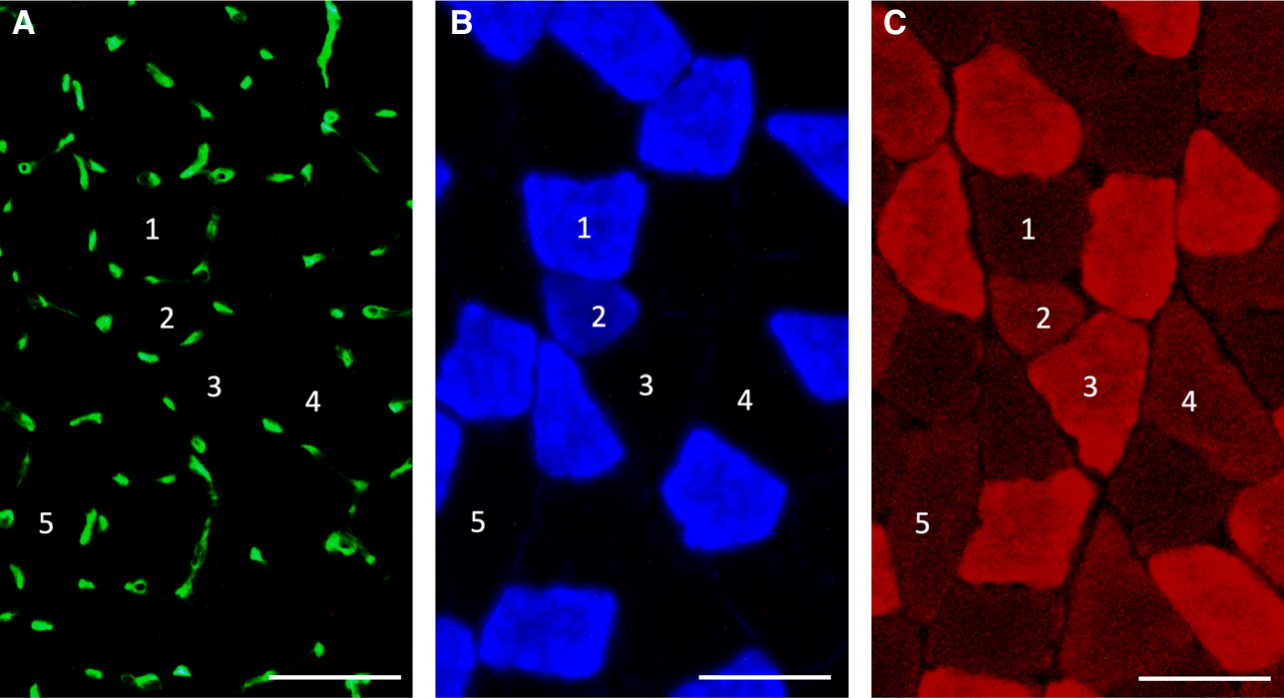
Representative immunofluorescent images of capillary (A) and type I (B) and type IIA (C) muscle fibers. 1, type I fiber; 2, type I + IIA fiber; 3, type IIA fiber; 4, type IIAX fiber; 5, type IIB + IIX fiber. Horizontal bars represent 50 *µ*m.

**Figure 4:**
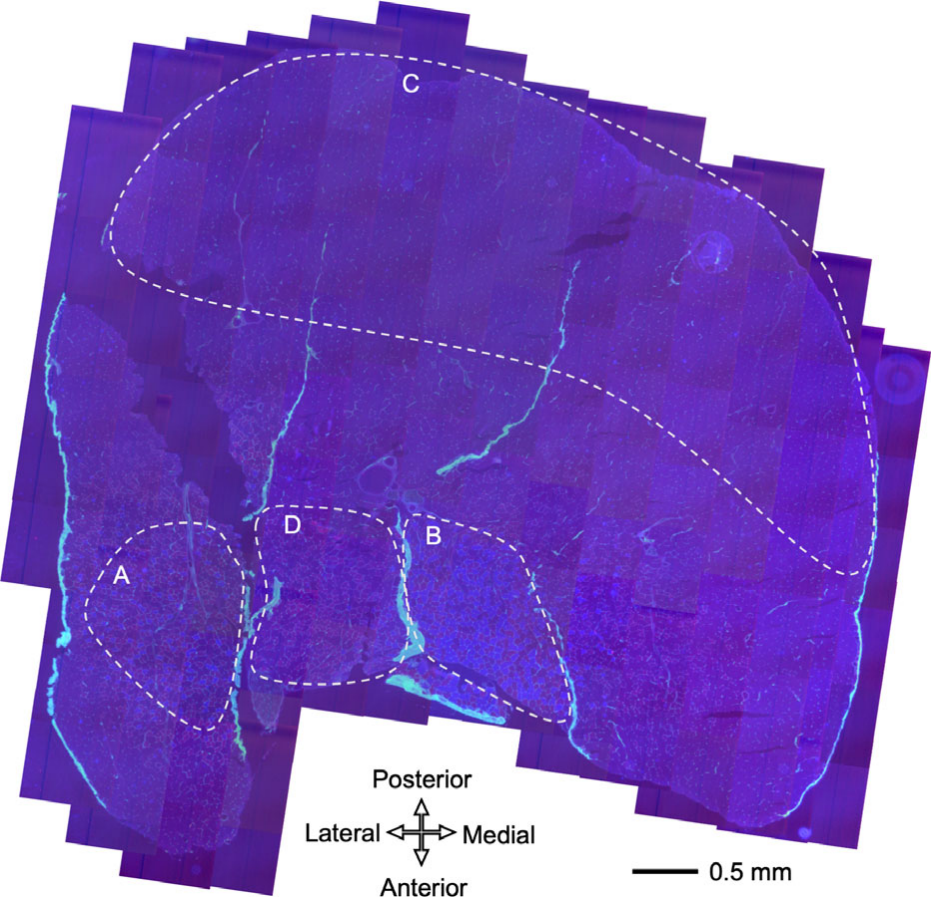
A representative merged fluorescent image obtained from the mid‐belly of gastrocnemius muscle. (A) GrL, (B) GrM, (C) Gw, (D) PL

Histochemical examinations of capillary profiles and muscle fiber phenotypes were conducted as previously reported, with slight modifications (Suzuki 2017). Briefly, 10‐*μ*m‐thick serial cross‐sections were obtained using a cryotome (CM‐1500; Leica Japan Inc., Tokyo, Japan) at −20°C. These sections were air dried, fixed with 100% ethanol at 4°C for 15 min, and then washed in 0.1 mol/L phosphate‐buffered saline with 0.05% Triton X‐100 (PBS‐T). Sections were then blocked with 10% normal goat serum at room temperature for 1 h and incubated at 4°C overnight with a mixture of fluorescein‐labelled Griffonia simplicifolia lectin [GSL I; FL 1101 (1:300); Vector Laboratories Inc., Burlingame, CA, USA], an anti‐type I myosin heavy chain (MHC) antibody (BA‐F8; mouse IgG2b; 1:100), and anti‐type IIA MHC antibody (SC‐71; mouse IgG1; 1:100) diluted with PBS‐T containing 5% goat normal serum. Sections were then reacted with a secondary antibody mixture containing Alexa Fluor 350‐labelled anti‐mouse IgG2b and Alexa Fluor 647‐labelled anti‐mouse IgG1 diluted with PBS‐T at room temperature for 1 h. Sections were coverslipped with Fluoromount/Plus (K048; Diagnostic BioSystems Co., Pleasanton, CA). Primary and secondary antibodies were purchased from the Developmental Studies Hybridoma Bank (University of Iowa) and Thermo Fisher Scientific Inc. (Tokyo, Japan), respectively. Fluorescent images of the incubated sections were observed using a microscope (Axio Observer; Carl Zeiss Japan, Tokyo, Japan). Muscle fiber phenotypes were classified as type I (blue), type I + IIA (faint blue and faint red), type IIA (red), type IIAX (faint red), and type IIB + IIX (unstained). Representative immunofluorescent images are shown in Figure 3. Nonoverlapping microscopic fields were selected at random from each muscle sample. The observer was blinded to the source (groups) of each slide during the measurements. Fluorescent images were obtained from SOL, PL, the lateral (GrL) and medial (GrM) portions of Gr, and Gw. The regions of GrL and GrM were identified as existence of type I fibers. Gw region was detected as existence of type IIB + IIX fibers only. Figure 4 represents a representative merged fluorescent image obtained from the mid‐belly of gastrocnemius muscle. Cross‐sectional area of Gw was the largest at the mid‐belly. The image does not show SOL, because cross‐sections obtained from approximately 5 mm distal part of the mid‐belly of gastrocnemius were used for SOL. The population of muscle fibers sampled from each muscle or muscle portion ranged from 200 to 300. The negative control without primary antibodies was confirmed to show no fluorescent signal.

A histochemical assessment for PGC‐1*α* expression in the nucleus was performed as reported previously (Suzuki 2019). Briefly, 10‐*μ*m‐thick cross‐sections were air dried, fixed with 3.8% formaldehyde in PBS for 15 min, and incubated at 4°C overnight with an Alexa Fluor 488‐labelled anti‐PGC‐1*α* antibody (1:500; Novus Biologicals, Centennial, CO) diluted with PBS‐T. After washing with PBS‐T, sections were reacted with 4′,6‐diamidino‐2‐phenylindole dihydrochloride (DAPI; D9542; Sigma‐Aldrich, St Louis, MO) to stain the nucleus fluorescent blue. Slides were then rinsed with distilled water and mounted as described above. Two individual images were obtained using the respective filter sets (filter set 49 or 38) in one microscopic field with a constant exposure time for each image and stored on a computer disk. In order to assess the expression of PGC‐1*α* in the nucleus, each RGB image was split into a green (PGC‐1*α*) or blue (nucleus) channel and converted to an eight‐bit greyscale image using ImageJ software (NIH, Bethesda, MD, USA). The co‐localized area of two images was obtained with a constant threshold value for each image. All fluorescent images were obtained within 24 h of staining.

### Biochemical analyses of enzyme activity

Frozen tissue powder was obtained using a frozen sample crusher (SK mill; Tokken Inc., Chiba, Japan) and homogenized with ice‐cold medium [10 mmol/L Hepes buffer, pH 7.4; 0.1% Triton X‐100; 11.5% (w/v) sucrose; and 5% (v/v) protease inhibitor cocktail (P2714; Sigma‐Aldrich)]. After centrifugation at 1500 g and 4°C for 10 min, the supernatant was used in enzyme activity analyses. The activities of 3‐hydroxyacyl‐CoA‐ dehydrogenase (HAD) and lactate dehydrogenase (LDH) were assayed according to the method of Bass et al. (1969). The activities of citrate synthase (CS) and phosphofructokinase (PFK) were assayed according to the methods of Srere (1969) and Passonneau and Lowry (1993), respectively. Pyruvate dehydrogenase complex (PDHc) activity was assayed according to the method of Ke et al. (2014). All measurements were conducted at 25°C with a spectrophotometer (U‐2001; Hitachi Co., Tokyo, Japan), and enzyme activities were obtained as micromoles per hour per milligram of protein. Total protein concentrations were measured using PRO‐MEASURE protein measurement solution (iNtRON Biotechnology Inc., Gyeonggi‐do, Korea).

### Western blot analyses

The tissue homogenates described in the previous section were used for Western blot analyses. A sample (50 *μ*g) was fractionated by SDS‐PAGE on 7.5 or 12% (w/v) polyacrylamide gels (TGX StainFree FastCast gel; Bio‐Rad Inc., Hercules, CA) and electrophoretically transferred to a polyvinylidene fluoride membrane. The bands in each lane on the membrane were detected with an ultraviolet imager (ChemiDoc; Bio‐Rad). The blots were blocked with 3% (w/v) bovine serum albumin, 1% (w/v) polyvinylpyrrolidone, and 0.3% (v/v) Tween‐20 in PBS for 1 h, and then exposed to a specific primary antibody (Santa Cruz Biotechnology Inc., Dallas, TX, USA) against Mfn2 (1:500; sc‐100560) or Drp1 (1:500; sc‐271583) diluted in PBS with 0.05% Tween‐20 for 1 h. After the blots had been incubated with an HRP‐labelled mouse IgG*κ* light chain binding protein (1:5,000; sc‐516102; Santa Cruz), they were reacted with Clarity Western ECL substrate (Bio‐Rad), and the required proteins were detected with an imager (ChemiDoc). The densities of the bands were normalized to the densities of all protein bands in each lane on the membrane (Gilda and Gomes 2013; Vigelsø et al. 2014). The densities of the bands were quantified using Image Lab software (Bio‐Rad) and normalized to the same sample that was run on every gel and transferred to every membrane.

### Statistical analyses

All values were log10‐transformed. Differences among four or five groups were examined using the Tukey‐Kramer multiple comparisons test. Differences in exercise performance before and after treadmill training with or without hyperbaric exposure were analyzed using Student's paired *t*‐test. The Pearson's product moment correlation coefficients (*r*) were used to establish correlations between the parameters observed in the present study. The effect sizes of r value were classified as small (*r* = 0.1), medium (*r* = 0.3), or large (*r* = 0.5). Mean, SD, and 95% confidence interval (CI) values are expressed after back‐transformation. Differences were considered significant at *P* < 0.05. When the CI values did not contain the zero value specified in the null hypothesis, differences were considered to be biologically important (du Prel et al. 2009). All statistical analyses were performed using EZR public domain software (Kanda 2013).

## Results

### Body and organ masses

Body weight was significantly lower in the four exercise‐trained groups than in the Sed group (Table 1, *P* < 0.05). The relative weights of the gastrocnemius, SOL, and PL were significantly higher in the four trained groups than in the Sed group (*P* < 0.05). The absolute and relative weights of the whole heart and left ventricle were significantly higher in the four trained groups than in the Sed group (*P* < 0.05). Thus, intermittent hypoxic exposure did not facilitate hypertrophy of the muscle and heart induced by exercise training.

**Table 1:** Body and organ masses.

	Sed (*n* = 10)	ET (*n* = 10)	H‐ET (*n* = 10)	HT (*n* = 10)	H‐HT (*n* = 10)
Body mass (g)	41.1 ± 2.5/2.4	37.5 ± 2.0/1.9^*a^	37.4 ± 1.5/1.5^*a^	37.5 ± 2.0/1.9^*a^	38.3 ± 2.9/2.7^*abcd^
Organ mass (mg)					
Gastrocnemius	166.3 ± 9.9/9.4	172.8 ± 6.9/6.7^a^	167.1 ± 12.3/11.5^ab^	172.8 ± 11.2/10.5^ac^	173.4 ± 11.2/101.5^ac^
Soleus	9.5 ± 0.68/0.64	10.3 ± 1.5/1.3^a^	10.7 ± 1.8/1.6^b^	10.4 ± 0.8/0.7^a^	10.4 ± 1.3/1.2^ac^
Plantaris	22.3 ± 2.9/2.6	24.6 ± 2.4/2.2^a^	23.7 ± 2.1/1.9^ab^	23.5 ± 2.3/2.1^ab^	23.9 ± 2.5/2.3^ab^
Whole heart	155.9 ± 8.8/8.3	180.9 ± 12.6/11.8^*a^	177.6 ± 12.1/11.4^*ab^	174.6 ± 15.8/14.5^*ab^	182.0 ± 12.8/12.0^*acd^
Left ventricle	112.3 ± 6.6/6.2	131.2 ± 9.5/8.9^*a^	130.2. ± 9.5/8.8^*ab^	129.7 ± 11.8/10.8^*a^	132.5 ± 11.2/10.3^*acd^
Organ mass‐to‐body mass ratio (mg g^−1^)					
Gastrocnemius	4.04 ± 0.28/0.26	4.60 ± 0.26/0.24^*a^	4.48 ± 0.21/0.20^*ab^	4.61 ± 0.37/0.35^*ac^	4.53 ± 0.32/0.29^*abd^
Soleus	0.23 ± 0.02/0.02	0.27 ± 0.04/0.03^*a^	0.29 ± 0.04/0.04^*ab^	0.28 ± 0.02/0.02^*ab^	0.27 ± 0.02/0.02^*acd^
Plantaris	0.54 ± 0.08/0.07	0.65 ± 0.07/0.06^*a^	0.63 ± 0.04/0.04^*ab^	0.63 ± 0.07/0.07^*a^	0.62 ± 0.07/0.06^*ab^
Whole heart	3.79 ± 0.15/0.15	4.82 ± 0.23/0.22^*a^	4.75 ± 0.21/0.20^*ab^	4.68 ± 0.29/0.27^*ac^	4.76 ± 0.21/0.20^*abd^
Left ventricle	2.75 ± 0.13/0.13	3.50 ± 0.19/0.18^*a^	3.48 ± 0.17/0.16^*a^	3.46 ± 0.23/0.22^*a^	3.46 ± 0.14/0.14^*ab^

Values are represented as means ± SD (upper/lower). *, Significantly different from the Sed group at *P < *0.05 using Tukey‐Kramer multiple comparison. a, b, c, and d, 95% confidence interval did not contain the parameter value specified in the null hypothesis between the Sed, ET, H‐ET, and HT groups, respectively.

### Maximal exercise capacity

Voluntary wheel training for 7 weeks markedly enhanced the maximal exercise capacity 5.0‐fold increase in total work (*P* < 0.001, Fig. 2A) and 2.4‐fold increase in run time to exhaustion (*P* < 0.001, Fig. 2B). ET for 4 weeks increased neither total work nor run time to exhaustion. A significant increase in total work (*P* = 0.008), but not run time, was observed after HT for 4 weeks. ET and HT with intermittent hypoxia significantly increased total work and run time values (*P* < 0.01). Total work and run time values were markedly greater in the H‐ET than ET groups (*P* = 0.093 and *P* = 0.065, respectively). Total work values were markedly greater in the H‐HT than ET (*P* = 0.002) and HT (*P* = 0.056) groups. Run time values were significantly greater in the H‐HT than ET groups (*P* = 0.003). Thus, short‐duration intermittent hypoxia had additive effects on exercise‐induced improvements in endurance exercise capacity in well‐trained mice.

### Metabolic enzyme activity

HAD activity levels were markedly higher in the H‐ET and H‐HT groups than in the Sed group in SOL and Gr (*P* < 0.05, Table 2). However, HAD activity levels were not markedly higher in the ET and HT groups than in the Sed group in SOL and Gr. In Gw, HAD activity levels were significantly higher in the four trained groups than in the Sed group (*P* < 0.05). CS activity levels in Gw were markedly higher in the H‐ET (*P* = 0.048) and H‐HT (*P* = 0.066) groups than in the Sed group. Moreover, in Gw, PDHc activity levels were significantly higher in the H‐ET group than in the Sed and ET groups (*P* < 0.05), and the values were significantly higher in the H‐HT group than in the Sed, ET, and HT groups (*P* < 0.001). PFK activity values in PL were significantly higher in the H‐HT group than in the HT group (*P* < 0.001). In DIA, CS activity levels were significantly higher in the HT and H‐HT groups than in the Sed group (*P* < 0.05). HAD activity values in DIA were significantly higher in the four trained groups than in the Sed groups (*P* < 0.001). Thus, training with intermittent hypoxic exposure enhanced enzyme activities associated with fatty acid oxidation in oxidative muscle and pyruvate oxidation in glycolytic muscle.

**Table 2:** Enzyme activity values (*µ*mol h^−1^ mg protein^−1^).

		Sed	ET	H‐ET	HT	H‐HT
CS	SOL	9.93 ± 2.7/2.1	10.4 ± 3.0/2.3	10.5 ± 2.3/1.9	10.8 ± 2.2/1.8	11.5 ± 2.3/1.9^a^
	PL	7.95 ± 0.9/0.8	9.84 ± 1.2/1.1^*a^	9.76 ± 1.2/1.1^*a^	9.20 ± 1.6/1.4^a^	9.19 ± 1.2/1.1^a^
	Gr	9.33 ± 1.6/1.4	12.1 ± 2.5/2.1^*a^	13.1 ± 1.6/1.5^*ab^	11.4 ± 2.5/2.0^ac^	12.9 ± 3.0/2.4^*a^
	Gw	3.83 ± 0.49/0.43	4.55 ± 0.73/0.63^a^	4.76 ± 0.55/0.49^*a^	3.98 ± 1.2/0.95^c^	4.71 ± 0.74/0.64^ad^
	DIA	16.5 ± 2.0/1.8	19.6 ± 2.3/2.0^a^	19.0 ± 3.2/2.7^a^	20.5 ± 4.0/3.3*^a^	20.7 ± 5.3/4.2^*a^
HAD	SOL	2.02 ± 0.46/0.38	2.36 ± 0.68/0.53	2.85 ± 0.60/0.50*ab	2.50 ± 0.80/0.60ac	3.07 ± 0.75/0.60*abd
	PL	1.51 ± 0.32/0.27	2.07 ± 0.26/0.23^*a^	2.11 ± 0.31/0.27^*a^	2.04 ± 0.27/0.24^*a^	1.93 ± 0.28/0.24^*a^
	Gr	1.61 ± 0.32/0.27	1.82 ± 0.46/0.37	1.95 ± 0.16/0.15^ab^	1.80 ± 0.29/0.25^ac^	1.95 ± 0.25/0.22^ad^
	Gw	0.58 ± 0.09/0.08a	0.78 ± 0.09/0.08^*^	0.77 ± 0.16/0.13^*a^	0.75 ± 0.19/0.15^*a^	0.76 ± 0.12/0.10^*a^
	DIA	8.43 ± 0.95/0.85	12.0 ± 1.8/1.6^*a^	11.5 ± 1.8/1.5^*a^	11.9 ± 1.4/1.2^*a^	12.0 ± 1.5/1.4^*a^
PDH	SOL	1.79 ± 0.46/0.36	1.53 ± 0.26/0.22^a^	1.47 ± 0.35/0.28^a^	1.42 ± 0.30/0.24^a^	1.46 ± 0.17/0.15^a^
	PL	1.20 ± 0.17/0.15	1.06 ± 0.09/0.09^a^	1.10 ± 0.13/0.11^a^	1.11 ± 0.19/0.16	1.19 ± 0.18/0.15
	Gr	1.24 ± 0.10/0.09	1.16 ± 0.13/0.11^*^	1.07 ± 0.16/0.14^*a^	1.13 ± 0.14/0.12^a^	1.08 ± 0.11/0.10^*a^
	Gw	1.04 ± 0.21/0.17	1.04 ± 0.17/0.15	1.30 ± 0.18/0.16^∫*ab^	1.12 ± 0.17/0.15^c^	1.57 ± 0.23/0.20^*¶abcd^
	DIA	3.17 ± 0.72/0.59	2.74 ± 0.36/0.32^a^	3.12 ± 0.35/0.32^b^	2.34 ± 0.41/0.35^*§abc^	2.78 ± 0.45/0.39^ad^
LDH	SOL	29.5 ± 15.1/10.0	14.8 ± 8.9/5.6^*a^	24.0 ± 10.3/7.2∫^b^	17.8 ± 6.4/4.7^*ac^	26.5 ± 12.6/8.5^∫bd^
	PL	113.5 ± 16.4/14.3	99.2 ± 10.3/9.3^a^	69.2 ± 15.7/12.8^ab^	84.0 ± 19.9/16.1^abc^	62.6 ± 14.2/11.6^abc^
	Gr	111.3 ± 9.6/8.9	81.6 ± 24.8/19.0^*a^	82.7 ± 16.1/13.5^*a^	87.8 ± 11.9/10.5^*a^	91.0 ± 13.0/11.4^*a^
	Gw	134.4 ± 16.1/14.4	128.3 ± 13.4/12.1	123.9 ± 23.0/19.4	116.1 ± 17.9/15.5^a^	139.7 ± 20.6/17.9^¶cd^
	DIA	57.4 ± 5.8/5.3	46.7 ± 6.4/5.6^*a^	45.7 ± 9.8/8.1^*a^	42.3 ± 6.5/5.6^*a^	51.8 ± 4.3/4.0^*abcd^
PFK	SOL	1.45 ± 0.89/0.55	1.47 ± 2.3/0.89	1.66 ± 4.4/1.2	1.40 ± 4.2/1.0	3.92 ± 5.3/2.2^abcd^
	PL	2.43 ± 1.8/1.0	2.24 ± 1.1/0.75	3.30 ± 2.5/1.4^b^	1.44 ± 1.5/0.73^§ac^	4.47 ± 2.4/1.6^¶abcd^
	Gr	1.24 ± 0.10/0.09	1.16 ± 0.13/0.11^*^	1.07 ± 0.16/0.14^*a^	1.13 ± 0.14/0.12^a^	1.08 ± 0.11/0.10^*a^
	Gw	8.42 ± 1.8/1.5	7.71 ± 3.5/2.4	8.37 ± 2.9/2.2	8.10 ± 2.1/1.7	8.02 ± 2.4/1.8
	DIA	7.74 ± 1.9/1.5	7.07 ± 1.5/1.2	7.67 ± 1.8/1.4	7.35 ± 2.0/1.6	8.13 ± 1.4/1.2^b^

Values are represented as means ± SD (upper/lower). *, ∫, §, and ¶, Significantly different from the Sed, ET, H‐ET, and HT groups, respectively, at *P* < 0.05 using the Tukey‐Kramer multiple comparisons test. a, b, c, and d, 95% confidence interval did not contain the parameter value specified in the null hypothesis between the Sed, ET, H‐ET and HT groups, respectively. CS, citrate synthase; HAD, 3‐hydroxyacyl‐CoA‐dehydrogenase; PDH, pyruvate dehydrogenase complex; LDH, lactate dehydrogenase; PFK, phosphofructokinase.

### Fiber‐type composition

The proportion of type I fibers was substantially greater in the H‐ET group than in the Sed group in PL (*P* = 0.085, Table 3). The proportion of type IIA and IIAX fibers was significantly greater in the four trained groups than in the Sed group (*P* < 0.05). The proportion of type IIB + IIX fibers was significantly lower in the four trained groups than in the Sed group in SOL, PL, and GrM (*P* < 0.05). Thus, short‐duration intermittent hypoxic exposure increased the proportion of highly oxidative muscle fibers in endurance‐trained hind limb muscles.

**Table 3:** Fiber type composition values (%).

	Type	Sed	ET	H‐ET	HT	H‐HT
SOL	I	46.2 ± 10.2/8.4	54.3 ± 8.6/7.5^a^	52.0 ± 9.3/7.9^ab^	52.8 ± 7.6/6.7^a^	55.7 ± 7.5/6.6^acd^
	I + IIA	0.16 ± 0.41/0.25	0.31 ± 0.64/0.36^a^	0.48 ± 0.62/0.38^ab^	0.38 ± 0.94/0.46^a^	0.26 ± 0.55/0.32^acd^
	IIA	46.3 ± 10.7/8.7	41.3 ± 7.1/6.1^a^	44.5 ± 10.4/8.4^b^	43.6 ± 8.5/7.1^ab^	41.0 ± 8.3/6.9^acd^
	IIAX	1.9 ± 3.7/1.4	1.2 ± 0.88/0.58^a^	0.93 ± 0.90/0.55^ab^	1.2 ± 1.2/0.71^ac^	1.2 ± 1.5/0.81^ac^
	IIB + IIX	2.5 ± 2.1/1.3	0.67 ± 1.9/0.73^*a^	0.28 ± 0.64/0.35^*ab^	0.34 ± 0.59/0.35^*ab^	0.27 ± 0.40/0.26^*ab^
PL	I	2.3 ± 5.5/1.9	4.3 ± 5.6/2.6^a^	6.9 ± 3.4/2.3^ab^	5.0 ± 5.1/2.6^ac^	2.2 ± 4.1/1.6b^cd^
	I + IIA	0.05 ± 0.12/0.10	0.14 ± 0.35/0.22^a^	0.18 ± 0.45/0.27^a^	0.16 ± 0.39/0.24^a^	0.14 ± 0.25/0.18^a^
	IIA	43.4 ± 9.2/7.6	54.9 ± 8.0/7.0^*a^	53.6 ± 6.4/5.7^*ab^	54.0 ± 6.0/5.4^*a^	52.1 ± 8.4/7.2^*abd^
	IIAX	5.3 ± 1.7/1.3	15.0 ± 5.1/3.9^*a^	16.0 ± 4.8/3.7^*ab^	16.7 ± 6.5/4.7^*ab^	16.8 ± 5.7/4.3^*abd^
	IIB + IIX	46.0 ± 8.1/6.9	22.1 ± 10.5/7.2^*a^	21.2 ± 6.9/5.2^*a^	21.2 ± 7.5/5.6^*a^	25.2 ± 12.2/8.3^*abcd^
GrL	I	17.8 ± 2.6/2.3	18.6 ± 3.2/2.7^a^	19.5 ± 2.8/2.5^ab^	19.3±2.7/2.4^ab^	19.7 ± 5.3/4.2^ab^
	I + IIA	0.04 ± 0.16/0.12	0.06 ± 0.25/0.17	0.09 ± 0.25/0.17^a^	0.04 ± 0.14/0.11^c^	0.04 ± 0.13/0.11^bc^
	IIA	47.3 ± 5.5/4.9	52.2 ± 7.1/6.2^a^	49.5 ± 4.8/4.4^ab^	52.4 ± 5.0/4.6^ac^	51.0 ± 4.0/3.7^abcd^
	IIAX	2.2 ± 5.4/1.8	10.3 ± 4.8/3.3^*a^	13.6 ± 5.8/4.1^*ab^	9.0 ± 9.0/4.6*^a^	7.4 ± 8.5/4.1^*abcd^
	IIB + IIX	30.5 ± 4.6/4.0	17.1 ± 4.8/3.7^*a^	15.3 ± 7.0/4.8^*ab^	14.7 ± 15.9/7.8^*ac^	17.6 ± 8.5/4.1^acd^
GrM	I	39.2 ± 9.8/7.9	43.6 ± 5.1/4.6^a^	44.3 ± 11.4/9.1^a^	42.9 ± 10.4/8.4^a^	40.6 ± 9.5/7.7^bcd^
	I + IIA	0.04 ± 0.14/0.11	0.18 ± 0.47/0.28^a^	0.04 ± 0.17/0.13^b^	0.08 ± 0.21/0.15^abc^	0.21 ± 0.62/0.33^acd^
	IIA	35.6 ± 9.1/7.3	46.1 ± 8.2/7.0^*a^	39.5 ± 6.9/5.9^ab^	46.8 ± 7.4/6.4^*a^	45.6 ± 4.7/4.3^*acd^
	IIAX	3.3 ± 4.7/2.1	4.0 ± 4.2/2.2^a^	8.1 ± 7.1/3.9^ab^	4.5 ± 3.5/2.1^abc^	4.7 ± 10.2/3.4^ac^
	IIB + IIX	18.2 ± 7.1/5.1	2.8 ± 5.8/2.1^*a^	4.7 ± 3.3/2.0^*ab^	2.3 ± 4.9/1.8^*ac^	3.1 ± 7.4/2.4^*acd^
Gw	IIX	100	100	100	100	100

Values are represented as means ± SD (upper/lower). *, Significantly different from the Sed group, respectively, at *P* < 0.05 using the Tukey‐Kramer multiple comparisons test. a, b, c, and d, 95% confidence interval did not contain the parameter value specified in the null hypothesis between the Sed, ET, H‐ET, and HT groups, respectively.

### Fiber cross‐sectional area

The fiber cross‐sectional area (FCSA) values of type I and IIA fibers in SOL were significantly higher in the H‐HT group than in the Sed group (*P* < 0.05, Table 4). The FCSA values for type IIAX fibers were significantly higher in the H‐HT group than in the other four groups (*P* < 0.01). The FCSA values of type IIA fibers in GrM were significantly higher in the H‐ET and H‐HT groups than in the Sed group (*P* < 0.05). Total FCSA values were markedly greater in the H‐HT group than in the Sed, ET, and HT groups in GrM. In SOL and GrL, total FCSA values were remarkably higher in the H‐HT group than in the other four groups. Thus, hybrid exercise training with intermittent hypoxic exposure facilitated exercise‐induced muscle hypertrophy.

**Table 4:** Fiber cross‐sectional area values (*µ*m^2^).

	Type	Sed	ET	H‐ET	HT	H‐HT
SOL	I	1461.9 ± 165.7/148.8	1636.1 ± 246.1/213.9^a^	1532.6 ± 256.3/219.6^ab^	1683.2 ± 214.1/189.9^abc^	1791.7 ± 373.6/309.1^*abcd^
	IIA	1544.3 ± 191.9/170.7	1700.2 ± 268.7/232.0^a^	1664.2 ± 255.6/221.6^a^	1680.9 ± 146.9/135.1^a^	1883.8 ± 365.5/306.1^*abcd^
	IIAX	1549.9 ± 284.8/240.6	1859.6 ± 335.0/283.9^a^	1943.4 ± 223.4/200.4^a^	1935.1 ± 260.4/229.5^ab^	2548.1 ± 534.8/442.1^*∫§¶abcd^
	Total	1506.9 ± 136.6/125.2	1675.5 ± 233.6/205.0^a^	1600.9 ± 225.9/198.0^a^	1684.5 ± 169.8/154.2^ac^	1842.2 ± 364.0/303.9^* abcd^
PL	I	962.9 ± 158.6/136.2	1217.9 ± 221.2/187.2^*a^	1131.6 ± 133.7/119.6^a^	1216.2 ± 250.7/207.8^*ac^	1131.2 ± 241.8/199.2^abd^
	IIA	1212.1 ± 153.2/136.0	1515.4 ± 310.9/258.0^*a^	1552.7 ± 145.3/132.9^*ab^	1473.7 ± 250.6/214.2^*a^	1369.0 ± 169.0/150.4^abcd^
	IIAX	1936.3 ± 307.4/265.3	2280.4 ± 409.9/347.4^a^	2429.5 ± 205.4/189.4^*ab^	2321.1 ± 352.1/305.7^*a^	2251.4 ± 324.2/283.4^ac^
	IIB + IIX	2596.3 ± 317.7/283.1	2703.9 ± 424.9/367.2^a^	2744.7 ± 308.1/277.0^a^	2730.3 ± 238.0/219.0^a^	2593.9 ± 668.4/531.5^c^
	Total	1715.1 ± 224.1/198.2	1855.9 ± 321.1/273.7^a^	1855.6 ± 176.9/161.5^a^	1836.8 ± 214.6/192.1^a^	1780.7 ± 250.4/219.5^a^
GrL	I	1453.5 ± 554.5/401.4	1474.8 ± 456.9/348.8	1417.2 ± 615.4/429.1	1447.3 ± 551.8/399.5	1695.3 ± 789.8/538.8^abcd^
	IIA	1492.5 ± 510.1/380.2	1600.5 ± 463.5/359.4^a^	1530.6 ± 700.7/480.7	1643.6 ± 532.1/402.0^ac^	1782.8 ± 673.6/488.9^abcd^
	IIAX	2121.0 ± 700.5/526.6	2488.9 ± 764.2/584.7^a^	2449.9 ± 1241.7/824.1^a^	2463.9 ± 810.3/609.8^a^	2456.9 ± 978.6/699.8^a^
	IIB + IIX	2901.2 ± 975.1/729.8	2940.9 ± 828.5/646.4	2927.9 ± 1470.7/978.9	2920.0 ± 682.0/552.9	3164.9 ± 972.6/744.0^acd^
	Total	1899.8 ± 589.36/449.8	1946.5 ± 524.6/413.2	1883.1 ± 904.1/610.8	1979.6 ± 624.9/475.0	2112.2 ± 792.7/576.8^abcd^
GrM	I	1596.0 ± 131.1/121.2	1710.0 ± 194.2/174.4^a^	1658.5 ± 212.1/188.0^a^	1631.0 ± 282.9/241.1^b^	1790.8 ± 310.0/264.2^abcd^
	IIA	1363.1 ± 135.1/122.9	1592.4 ± 228.3/199.6^a^	1622.3 ± 184.5/165.7^*ab^	1516.5 ± 210.3/184.7^abc^	1676.1 ± 311.2/262.5^*abcd^
	IIAX	1790.9 ± 151.5/139.7	2088.9 ± 344.9/296.0^a^	2088.0 ± 303.0/264.6^a^	1974.7 ± 331.5/283.8^abc^	2038.3 ± 353.6/301.3^a^
	IIB + IIX	2071.4 ± 156.3/145.3	2298.0 ± 638.9/499.9^a^	2316.2 ± 239.5/217.0^a^	2087.3 ± 444.8/366.7^bc^	2220.6 ± 232.3/210.3^ad^
	Total	1616.8 ± 75.4/72.0	1722.8 ± 207.6/185.3^a^	1769.4 ± 190.0/171.6^ab^	1620.3 ± 196.2/175.0^c^	1818.4 ± 307.4/262.9^abd^
Gw	IIB + IIX	2650.4 ± 457.0/389.8	2714.7 ± 274.6/249.4^a^	2757.8 ± 204.3/190.2^ab^	2851.5 ± 259.3/237.7^abc^	2647.1 ± 234.5/215.4^cd^

Values are represented as means ± SD (upper/lower). *, ∫, §, and ¶, Significantly different from the Sed, ET, H‐ET, and HT groups, respectively, at *P* < 0.05 using the Tukey‐Kramer multiple comparisons test. a, b, c, and d, 95% confidence interval did not contain the parameter value specified in the null hypothesis between the Sed, ET, H‐ET and HT groups, respectively.

### PGC‐1*α* expression

**Figure 5:**
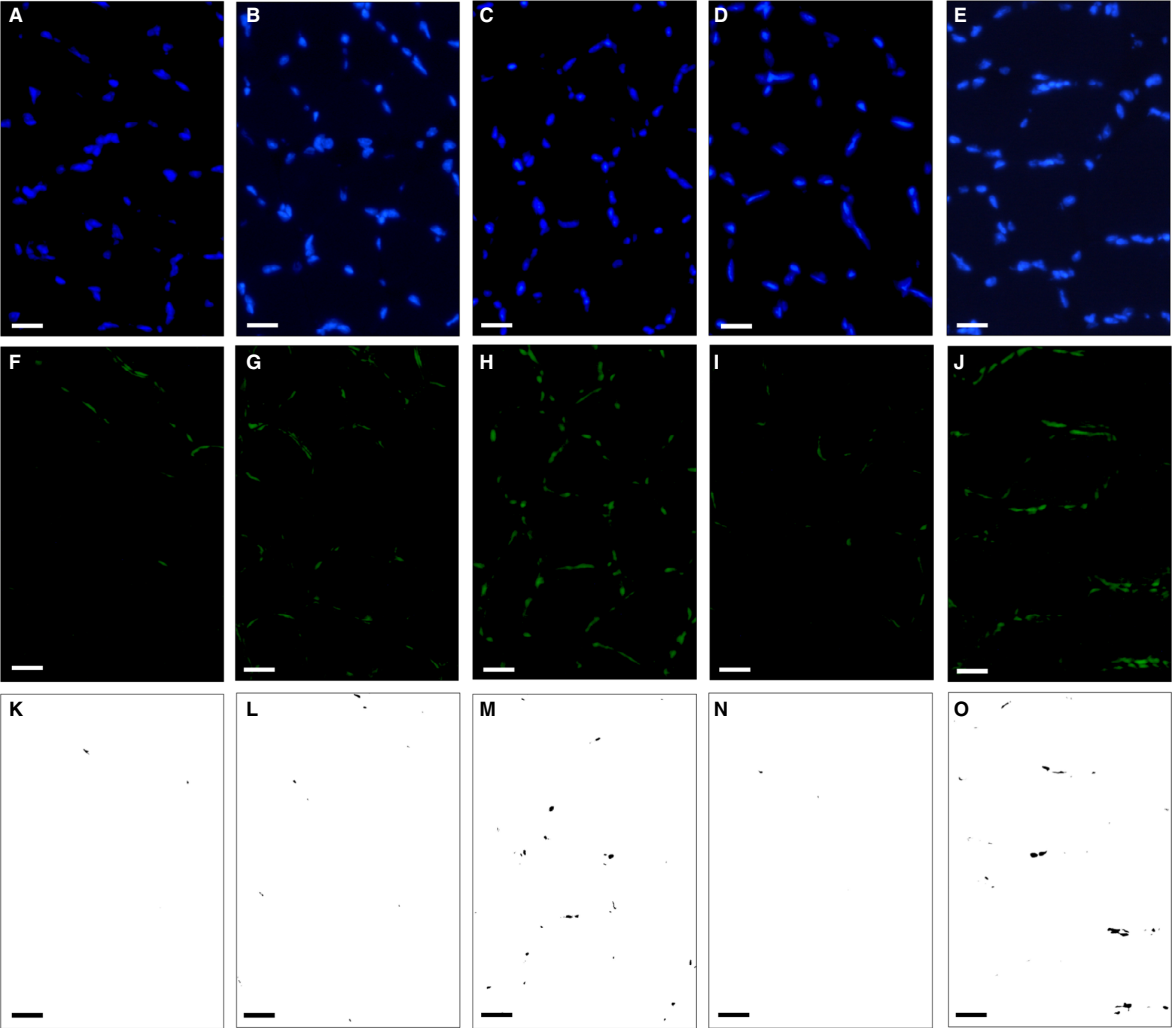
Representative immunofluorescent images of the soleus muscle. (A–E) Immunofluorescent images of the nucleus. (F–J) Immunofluorescent images of PGC‐1*α*. (K–O) Images of co‐localized area. (A, F, and K) Sed group. (B, G, and L) ET group. (C, H, and M) H‐ET group. (D, I, and N) HT group. (E, J, and O) H‐HT group. Scale bars represent 20 *μ*m.

**Figure 6:**
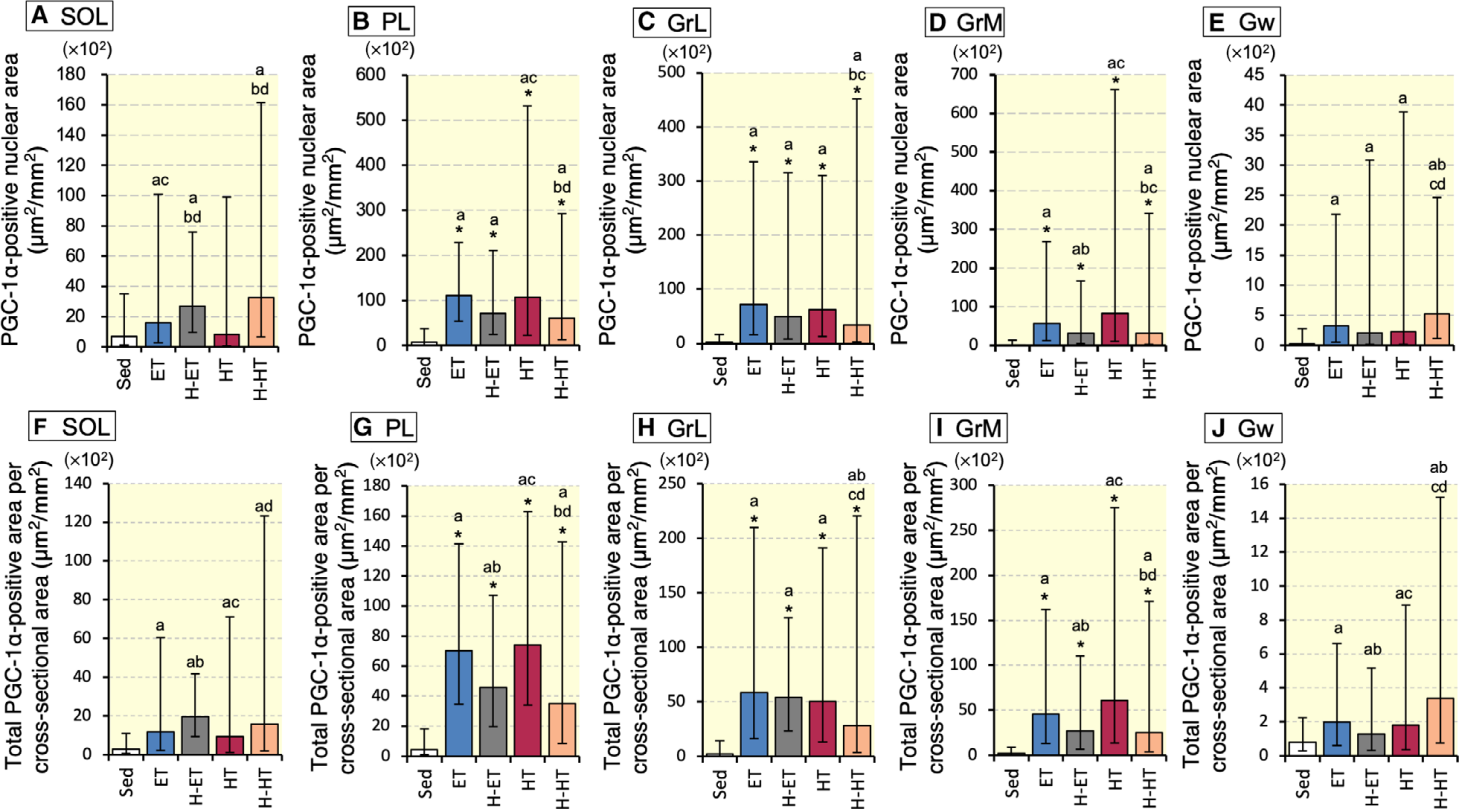
Histochemical identification of PGC‐1*α* expression in the nucleus. (A‐E) Co‐localized area values for PGC‐1*α* and the nucleus. (F‐J) Total PGC‐1*α* positive area per muscle cross‐sectional area. Values are represented as means ± SD. SOL, soleus muscle; PL, plantaris muscle; GrL, red portion of lateral gastrocnemius muscle; GrM, red portion of medial gastrocnemius muscle; Gw, white portion of gastrocnemius muscle. *Significantly different from the Sed group at *P* < 0.05 using the Tukey‐Kramer multiple comparisons test. a, b, c, and d, the 95% confidence interval did not contain the parameter value specified in the null hypothesis between the Sed, ET, H‐ET, and HT groups, respectively.

Representative fluorescent images in SOL are shown in Figure 5(A–O). The PGC‐1*α*‐positive nuclear area was substantially greater in the H‐ET (CI: 1.31–2.16, *P* = 0.97 and CI: 2.54–4.17, *P* = 0.62, respectively) and H‐HT (CI: 1.29–3.15, *P* = 0.93 and CI: 2.50–6.07, *P* = 0.51, respectively) groups than in the ET and HT groups in SOL (Fig. 6A). Total PGC‐1*α* levels were also considerably greater in the H‐ET group (CI: 1.39–2.01, *P* = 0.97 and CI: 1.78–2.56, *P* = 0.86, respectively) than in the ET and HT groups, and were greater in H‐HT group (CI: 1.10–2.67, *P* = 0.95) than in HT group in SOL (Fig. 6F). In Gw, total and nuclear PGC‐1*α* levels were substantially greater in the H‐HT group (CI: 1.29–2.67, *P* = 0.86, and CI: 1.62–3.42, *P* = 0.93, respectively) than in the HT group (Fig. 6J). Although a statistical significance was not detected, additive effects induced by intermittent hypoxia were evaluated by the CI values in SOL and Gw. Thus, short‐duration intermittent hypoxia modestly facilitated the exercise‐induced expression of PGC‐1*α* protein in highly oxidative muscle and glycolytic muscle portions.

### Protein expression

**Figure 7:**
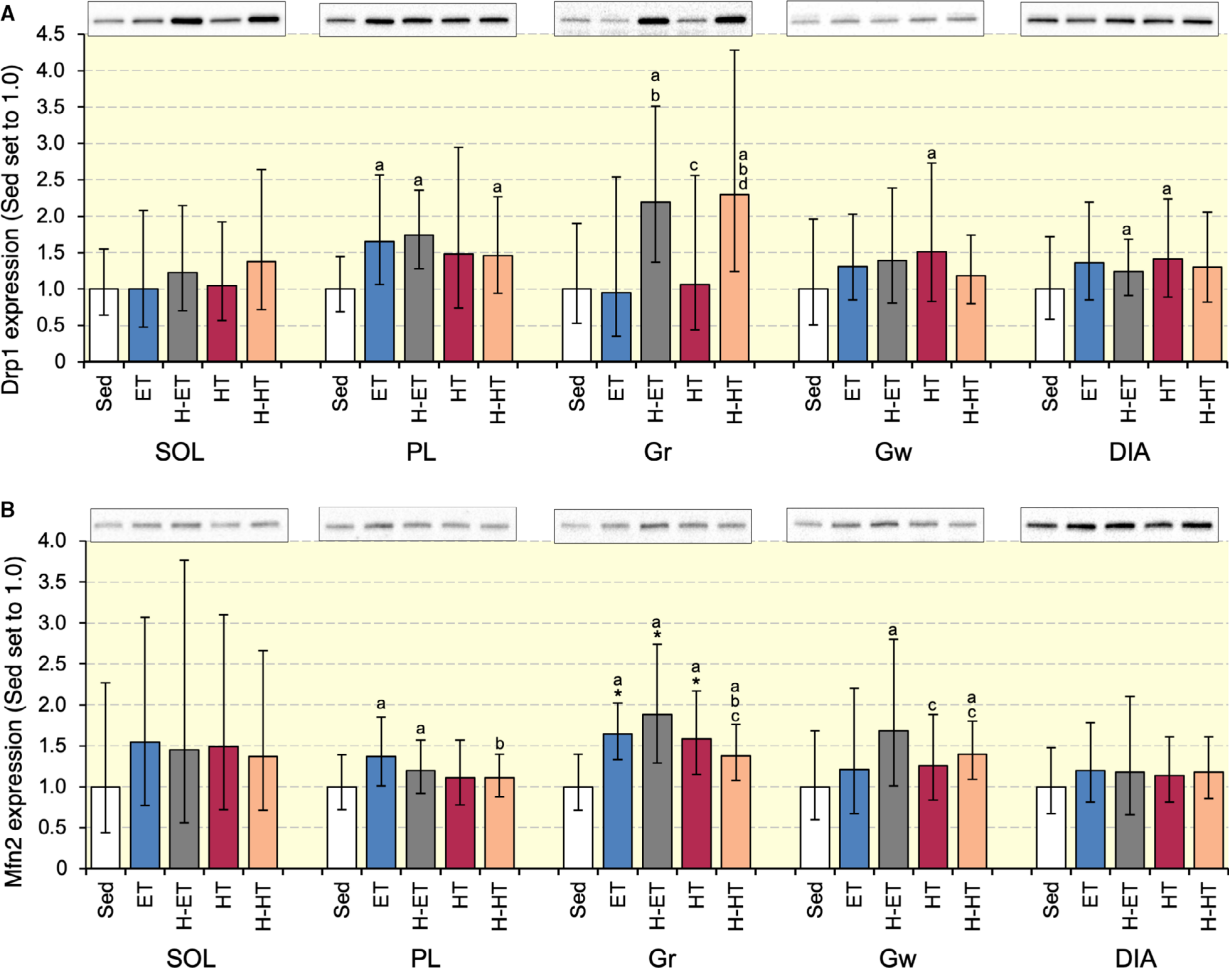
Expression of Drp1 (A) and Mfn2 (B) proteins after 4 weeks of exercise training with and without intermittent hypoxia. Values are represented as means ± SD. *, significantly different at *P* < 0.05 using the Tukey‐Kramer multiple comparisons test. a, b, c, and d, the 95% confidence interval did not contain the parameter value specified in the null hypothesis between the Sed, ET, H‐ET, and HT groups, respectively.

Drp1 protein expression in Gr was substantially stronger in the H‐ET (CI: 1.59–3.01, *P* = 0.15) and H‐HT (CI: 1.50–3.50, *P* = 0.11) groups than in the Sed group (Fig. 7A). Moreover, Drp1 levels were considerably greater in the H‐ET (CI: 1.66–3.15, *P* = 0.11 and CI: 1.50–2.85, *P* = 0.20, respectively) and H‐HT (CI: 1.58–3.67, *P* = 0.080 and CI:1.42–3.32, *P* = 0.15, respectively) groups than in the ET and HT groups. Mfn2 protein expression in Gr was markedly stronger in the ET, H‐ET, and HT than in the Sed group (*P* < 0.05, Fig. 7B). Mfn2 levels were markedly higher in the H‐ET and H‐HT groups than in the Sed group in Gw. Thus, short‐duration intermittent hypoxia substantially up‐regulates Drp1 protein in hind limb muscles.

### Capillarization

The capillary‐to‐fiber (C:F) ratio and capillary density values were significantly greater in the four trained groups than in the Sed group in PL (*P* < 0.05, Table 5). In SOL and GrL, C:F values were substantially higher in the H‐HT group than in the Sed group (*P* = 0.083 and *P* = 0.095, respectively). Thus, daily short‐duration intermittent hypoxic exposure facilitated exercise‐induced capillary growth in highly oxidative muscle of hybrid‐trained mice.

**Table 5:** Capillary‐to‐fiber ratio and capillary density values.

	Sed	ET	H‐ET	HT	H‐HT
Capillary‐to‐fiber ratio					
SOL	1.86 ± 0.15/0.17	1.96 ± 0.12/0.13^a^	1.88 ± 0.13/0.14^b^	1.96 ± 0.20/0.21^ac^	2.07 ± 0.16/0.17^abcd^
PL	1.73 ± 0.16/0.17	2.18 ± 0.12/0.13^*a^	2.19 ± 0.13/0.14^*a^	2.12 ± 0.21/0.22^*abc^	2.15 ± 0.19/0.21^*a^
GrL	2.20 ± 0.21/0.22	2.28 ± 0.23/0.25^a^	2.34 ± 0.23/0.25^a^	2.29 ± 0.30/0.34^a^	2.51 ± 0.31/0.34^abcd^
GrM	2.03 ± 0.19/0.20	2.06 ± 0.20/0.22	2.10 ± 0.15/0.16^a^	2.19 ± 0.30/0.33^a^	2.07 ± 0.23/0.26^abcd^
Gw	1.03 ± 0.11/0.12	0.97 ± 0.17/0.19^a^	0.98 ± 0.15/0.17^a^	1.12 ± 0.20/0.23^abc^	1.04 ± 0.16/0.17^bcd^
Capillary density (number mm^−2^)					
SOL	1251.5 ± 63.0/66.4	1205.7 ± 141.5/160.3^a^	1241.4 ± 121.8/135.1^b^	1212.1 ± 110.6/121.8^ac^	1168.6 ± 153.5/176.7^acd^
PL	1039.5 ± 124.0/140.8	1303.0 ± 129.8/144.1^*a^	1286.2 ± 108.9/119.0^*a^	1240.5 ± 121.5/134.7^*abc^	1283.6 ± 187.8/220.0^*ad^
GrL	1159.9 ± 266.9/346.6	1204.4 ± 240.4/300.3	1285.6 ± 337.2/457.0	1206.1 ± 229.0/282.7^bc^	1161.6 ± 290.4/387.2^abc^
GrM	1214.4 ± 142.4/161.3	1232.5 ± 145.1/164.4	1306.8 ± 99.1/107.3	1330.2 ± 146.9/165.2^bc^	1162.9 ± 212.3/259.6^abc^
Gw	490.0 ± 53.0/59.4	434.9 ± 36.1/73.8^a^	452.4 ± 71.5/84.8^a^	485.2 ± 76.3/90.5^bc^	478.9 ± 68.4/79.7^bc^

Values are represented as means ± SD (upper/lower). *, Significantly different from the Sed group at *P* < 0.05 using the Tukey‐Kramer multiple comparisons test. a, b, c, and d, 95% confidence interval did not contain the parameter value specified in the null hypothesis between the Sed, ET, H‐ET, and HT groups, respectively.

### Correlation analysis

A positive correlation was found between HAD levels and maximal work values in Gr and SOL (Table 6). CS levels positively correlated with maximal work values in Gr and Gw. A positive correlation was observed between PDHc levels and maximal work values in Gw. Expression levels of PGC‐1*α* in the nucleus positively correlated with maximal work values in Gw. There was a positive correlation between total FCSA and maximal work values in GrM and SOL. C:F ratio values positively correlated with maximal work and nuclear PGC‐1*α* levels in SOL. A small positive correlation, but not significant, was found between DRP1 levels and maximal work values in Gr and SOL.

**Table 6:** Correlations.

	Explanatory variable	Response variable	*r*	*P* value
Gr				
HAD		Maximal work	0.345	0.015
CS		Maximal work	0.439	0.0016
DRP1		Maximal work	0.265	0.065
Total FCSA (GrM)		Maximal work	0.284	0.048
C:F ratio (GrL)		Maximal work	0.274	0.056
Gw				
PDHc		Maximal work	0.421	0.0026
CS		Maximal work	0.389	0.0057
PGC‐1*α* (nuclear)		Maximal work	0.413	0.0040
PGC‐1*α* (total)		Maximal work	0.253	0.087
SOL				
HAD		Maximal work	0.424	0.0024
CS		Maximal work	0.220	0.13
DRP1		Maximal work	0.270	0.061
Total FCSA		Maximal work	0.314	0.032
C:F ratio		Maximal work	0.314	0.032
PGC‐1*α* (nuclear)		C:F ratio	0.30	0.045
PGC‐1*α* (total)		C:F ratio	0.238	0.057

*r*, Pearson's product moment correlation coefficient.

## Discussion

The main result of the present study was that, in mice that have been trained from an early age, short‐duration intermittent hypoxic exposure facilitated endurance exercise performance via promoted fatty acid oxidation in oxidative muscle portion and pyruvate oxidation in glycolytic muscle portion. In some cases, synergistic effects of intermittent hypoxic exposure and exercise training observed in the present study were evaluated by the CI values as a biologically important difference (du Prel et al. 2009). In highly trained mice, substantial, but not statistically significant, changes in variables concerning muscle metabolism and morphology probably enhance endurance capacity in an integrated manner. Further studies on trained human subjects are needed to clarify whether exercise performance in athletes is promoted by intermittent hypoxic exposure.

Intermittent hypoxia used in the present study as well as 1‐h acute endurance exercise markedly enhanced erythropoietin mRNA, one of the HIF‐1*α* target genes, in the kidney only immediately posttreatment, indicating that HIF‐1*α* was transiently stabilized (Suzuki 2016). However, in the red gastrocnemius, PGC‐1*α* mRNA expression was significantly increased after 6 h of acute intermittent hypoxia and after 3 weeks of exercise training with intermittent hypoxia (Suzuki 2016). PGC‐1*α* mRNA expression was shown to markedly increased after acute exercise and then reduced within 10 h in untrained mice (Suzuki 2016) and within 19 h in untrained humans (Egan et al. 2010). In the present study, 48 h after the last exhaustive exercise, protein levels of PGC‐1*α* were markedly enhanced in four exercise groups (Fig. 6). Although intermittent hypoxia slightly facilitated PGC‐1*α* levels in SOL and Gw, a significant positive correlation was observed between nuclear PGC‐1*α* levels and maximal work values in Gw (Table 6). PGC‐1*α* was shown to induce pyruvate dehydrogenase kinase (PDK)‐4 gene expression, thus inhibiting PDHc, in vitro (Wende et al., 2007). Endurance training for 4 weeks was shown to markedly decrease PDHc activity in rat gastrocnemius muscle (Nakai et al. 2002). In the present study, marked reduction in PDHc levels were observed in four exercise groups in Gr (Table 2). In contrast, in Gw, ET and HT with intermittent hypoxia significantly enhanced PDHc and CS activity levels. Moreover, these activity levels were significantly correlated with maximal work values (Table 6). These findings may indicate that PGC‐1*α* inhibited PDHc activity in oxidative muscle portions but did not in glycolytic muscle portion. This notion may be explained by the absolute expression levels of PGC‐1*α* protein, which were approximately 15 times greater in oxidative muscle portions than in glycolytic muscle portion (Fig. 6). Muscle‐specific PGC‐1*α* overexpression has been shown to facilitate post‐exercise glucose uptake and glycogen replenishment in skeletal muscle (Wende et al. 2007). Enhanced PGC‐1*α* expression levels observed after 48 h of last exercise bout may indicate that post‐exercise glycogen replenishment was facilitated. Moreover, cross‐sectional area of Gw was the largest in the mice triceps surae muscle (Fig. 4). Thus, in glycolytic muscle portion, promoted pyruvic acid oxidation induced by daily intermittent hypoxic exposure may contribute to enhance endurance capacity in well‐trained mice.

In untrained adult mice, endurance training with intermittent hypoxia for 3 weeks was shown to significantly enhance HAD levels in Gr (Suzuki, 2016). In the present study, using well‐trained mice, HAD activity levels in SOL and Gr were markedly increased after ET and HT with intermittent hypoxia (Table 2) and were significantly correlated with maximal work values (Table 6). Thus, intermittent hypoxia may promote endurance capacity via facilitating fatty acid oxidation in oxidative muscle portion in well‐trained mice.

Exercise was shown to affect mitochondrial fusion and fission in skeletal muscle. In well‐trained mice, as used in the present study, endurance training increased Drp1 protein levels, while sprint interval training increased both Drp1 and Mfn2 expression levels (Suzuki 2019). In that study and the present study, the organs were harvested 48 h after the last exercise bout. Therefore, changes in protein expression represented chronic adaptive changes induced by exercise training. Muscle‐specific Drp1 heterozygote (mDrp1^+/−^) mice showed a significantly lower maximal running speed than wild type mice (Moore et al. 2019). Drp1 inhibition was shown to suppress mitochondrial respiration and reactive oxygen species (ROS) without morphological changes in mitochondria, that is, fission, in rat cardiomyocyte (Zhang et al, 2017). Unfortunately, in the present study, a weak positive correlation was observed between Drp1 protein levels and maximal work values (Table 6). Thus, Drp1 protein levels may moderately contribute to enhanced endurance performance.

In the present study, total work values were considerably greater in H‐HT than in H‐ET groups (Fig. 2). LDH and PDHc activity levels in Gw showed substantially greater values in H‐HT than H‐ET groups (Table 2). In SOL and PL, PFK levels in H‐HT group were also considerably greater than those in H‐ET group. Thus, H‐HT may improve glucose metabolism in both oxidative and glycolytic muscle portions. In the present study, total FCSA values were remarkably increased by H‐HT in SOL, GrL, and GrM. A significant positive correlation was observed between total FCSA and maximal work values in SOL and GrM (Table 6). Acute combined exercise consisting of high‐intensity interval and endurance exercise markedly up‐regulated mRNA levels of myogenic regulatory factor‐4, but respective, single, uncombined regimens did not (Skovgaard et al. 2016). Although further investigations are needed to elucidate the mechanisms, H‐HT may facilitate muscle hypertrophy in oxidative muscle portion. In SOL, C:F ratio values were substantially greater in H‐HT than in H‐ET group (Table 5) and the values showed significant positive correlation with maximal work values and nuclear PGC‐1*α* levels (Table 6). PGC‐1*α* has been shown to facilitate exercise‐induced capillary growth in skeletal muscle (Leick et al. 2009). Thus, H‐HT may facilitate capillary growth in oxidative fiber‐rich muscle, thereby increasing the supply of oxygen and metabolic substrates during exercise. The absolute work on the last day of treadmill training was markedly lower with hybrid exercise (33.6 J (g body weight)^−1^) than with endurance exercise (48.2 J (g body weight)^−1^). Thus, H‐HT might be a beneficial strategy for facilitating muscle glucose metabolism, fiber hypertrophy, and capillarity, thereby enhancing endurance capacity.

## Conflict of Interest

None declared.
